# Neonatal Development in Prenatally Zika Virus-Exposed Infant Macaques with Dengue Immunity

**DOI:** 10.3390/v13091878

**Published:** 2021-09-20

**Authors:** Karla Ausderau, Sabrina Kabakov, Elaina Razo, Ann M. Mitzey, Kathryn M. Bach, Chelsea M. Crooks, Natalie Dulaney, Logan Keding, Cristhian Salas-Quinchucua, Lex G. Medina-Magües, Andrea M. Weiler, Mason Bliss, Jens Eickhoff, Heather A. Simmons, Andres Mejia, Kathleen M. Antony, Terry Morgan, Saverio Capuano, Mary L. Schneider, Matthew T. Aliota, Thomas C. Friedrich, David H. O’Connor, Thaddeus G. Golos, Emma L. Mohr

**Affiliations:** 1 Department of Kinesiology, Occupational Therapy Program, University of Wisconsin–Madison, Madison, WI 53706, USA; kausderau@wisc.edu (K.A.); kabakov@wisc.edu (S.K.); kmbach3@wisc.edu (K.M.B.); ndulaney@wisc.edu (N.D.); mlschnei@wisc.edu (M.L.S.); 2 Waisman Center, University of Wisconsin–Madison, Madison, WI 53706, USA; 3 Department of Pediatrics, School of Medicine and Public Health, University of Wisconsin–Madison, Madison, WI 53792, USA; razo2@wisc.edu; 4 Department of Comparative Biosciences, University of Wisconsin–Madison, Madison, WI 53706, USA; amitzey@wisc.edu (A.M.M.); lkeding@wisc.edu (L.K.); golos@primate.wisc.edu (T.G.G.); 5 Department of Pathobiological Sciences, University of Wisconsin–Madison, Madison, WI 53706, USA; ccrooks@wisc.edu (C.M.C.); cristhian.salas@wisc.edu (C.S.-Q.); medinamagues@wisc.edu (L.G.M.-M.); tfriedri@wisc.edu (T.C.F.); 6 Wisconsin National Primate Research Center, University of Wisconsin–Madison, Madison, WI 53715, USA; amweiler@wisc.edu (A.M.W.); mibliss@wisc.edu (M.B.); hsimmons@primate.wisc.edu (H.A.S.); amejia@primate.wisc.edu (A.M.); capuano@wisc.edu (S.C.III); dhoconno@wisc.edu (D.H.O.); 7 Department of Biostatistics and Medical Informatics, University of Wisconsin–Madison, Madison, WI 53792, USA; jceickhoff@wisc.edu; 8 Department of Obstetrics and Gynecology, University of Wisconsin–Madison, Madison, WI 53705, USA; kantony@wisc.edu; 9 Center for Developmental Health, Department of Obstetrics and Gynecology, Knight Cardiovascular Institute, Oregon Health & Science University, Portland, OR 97239, USA; morgante@ohsu.edu; 10 Department of Veterinary and Biomedical Sciences, University of Minnesota, Minneapolis, MN 55108, USA; mtaliota@umn.edu; 11 Department of Pathology and Laboratory Medicine, University of Wisconsin–Madison, Madison, WI 53705, USA

**Keywords:** macaque model, prenatal ZIKV exposure, neurodevelopment, maternal DENV infection

## Abstract

Infants exposed to Zika virus (ZIKV) prenatally may develop birth defects, developmental deficits, or remain asymptomatic. It is unclear why some infants are more affected than others, although enhancement of maternal ZIKV infection via immunity to an antigenically similar virus, dengue virus (DENV), may play a role. We hypothesized that DENV immunity may worsen prenatal ZIKV infection and developmental deficits in offspring. We utilized a translational macaque model to examine how maternal DENV immunity influences ZIKV-exposed infant macaque neurodevelopment in the first month of life. We inoculated eight macaques with prior DENV infection with ZIKV, five macaques with ZIKV, and four macaques with saline. DENV/ZIKV-exposed infants had significantly worse visual orientation skills than ZIKV-exposed infants whose mothers were DENV-naive, with no differences in motor, sensory or state control development. ZIKV infection characteristics and pregnancy outcomes did not individually differ between dams with and without DENV immunity, but when multiple factors were combined in a multivariate model, maternal DENV immunity combined with ZIKV infection characteristics and pregnancy parameters predicted select developmental outcomes. We demonstrate that maternal DENV immunity exacerbates visual orientation and tracking deficits in ZIKV-exposed infant macaques, suggesting that human studies should evaluate how maternal DENV immunity impacts long-term neurodevelopment.

## 1. Introduction

Prenatal Zika virus (ZIKV) exposure results in a spectrum of abnormalities that includes birth defects and neurodevelopmental deficits. Nearly 30% of ZIKV-exposed infants are asymptomatic at birth and manifest neurodevelopmental deficits in early childhood [1,2]. These neurodevelopmental abnormalities involve language, cognitive, motor, hearing, and visual deficits [2,3,4], along with diminished mobility, communication, and social cognition [1,3]. Only 10% of infants have defects apparent at birth, termed congenital Zika syndrome, which includes ocular anomalies, brain anomalies, cranial dysmorphologies, congenital contractures and hearing loss [5]. Currently, there is little understanding of how specific characteristics of maternal ZIKV infection impact infant developmental outcomes, particularly as it relates to a maternal history of previous infections such as dengue virus (DENV).

The impact of previous DENV immunity on maternal ZIKV infection and infant developmental outcomes has not yet been defined. The presence of DENV IgG during ZIKV infection worsens ZIKV infection outcomes in some scenarios, i.e., in cell culture and murine models [6]. In non-pregnant macaque studies, the presence of DENV IgG does not increase ZIKV titers or disease [7,8]. Human pregnancy studies have begun teasing apart this question by examining pregnancies with prenatal ZIKV exposure and DENV immunity, and defining rates of miscarriage, abnormal infant physical exams, congenital Zika syndrome, and abnormal infant neuroimaging [9]. The role of maternal DENV immunity on prenatal ZIKV infection varies between the populations studied, with some studies identifying no change in the rates of miscarriage and microcephy [10,11,12], and others finding that maternal DENV immunity may reduce the risk of congenital Zika syndrome [13]. However, none of these studies have begun to elucidate how infant neurodevelopment is impacted by prenatal ZIKV infection in mothers who have a history of DENV infection. Defining the impact of maternal pre-existing DENV immunity on prenatal ZIKV infection is critical to identifying children who are at high risk of deficits early and intervening to maximize functional outcomes.

Translational animal models of prenatal ZIKV infection are necessary to define how infant neurodevelopmental outcomes differ in pregnancies with and without pre-existing DENV immunity. Early murine studies of maternal ZIKV infection in animals with pre-existing DENV immunity found that offspring of DENV/ZIKV-exposed pregnancies had worse visual impairment than ZIKV-exposed pups [14]. However, significant limitations exist with murine models of congenital ZIKV infection because of different types of placentation and shorter duration of pregnancy [15,16]. Preclinical macaque models can complement human clinical studies by identifying correlates of developmental outcomes that can be translated to human studies. We have previously described a macaque model for defining the pathogenesis of developmental deficits in ZIKV-exposed infants with no apparent birth defects found in the first seven days of life [17]. Rhesus macaque models more closely resemble human fetal brain development and infant development than other animal models [15], and have been used to model human infant development for decades [18], which positions them as the ideal model in which to define how prior maternal DENV infection and prenatal ZIKV infection impact neonatal development. Careful control of pre-existing DENV immunity with a single DENV serotype and timing it with gestational ZIKV infection at early pregnancy is critical for defining how DENV immunity impacts infant development.

In this manuscript, we define the developmental outcomes of infant macaques exposed to ZIKV prenatally with and without maternal DENV immunity and identify the maternal ZIKV infection and fetal growth factors that significantly predict neurodevelopment. We describe the features of maternal ZIKV infection in the dams with and without DENV immunity, including maternal ZIKV viremia, maternal–fetal interface tissue pathology and virus distribution, fetal growth, neonatal health, and growth. This is the first comparison of development outcomes between prenatally ZIKV-exposed infant macaques with and without pre-existing DENV immunity, and the first to identify significantly associated maternal ZIKV infection characteristics that influence developmental outcomes in a multivariate model.

## 2. Materials and Methods

### 2.1. Study Design

Indian-origin rhesus macaques (*Macaca mulatta*) were inoculated with ZIKV or phosphate-buffered saline (PBS) during the first trimester (term is 165 ± 10 days) (Table 1). All dams were part of the Specific Pathogen Free (SPF) colony at the Wisconsin National Primate Research Center (WNPRC) and were free of *Macacine herpesvirus 1* (Herpes B), simian retrovirus type D (SRV), simian T-lymphotropic virus type 1 (STLV), and simian immunodeficiency virus (SIV). The pregnancies of the 8 DENV/ZIKV and 4 of 5 ZIKV pregnancies have been described earlier [19]. All infant studies, along with the pregnancies of all control dams and ZIKV dam 044-109, are presented for the first time in this report.

### 2.2. Ethics Statement

All monkeys are cared for by the staff at the WNPRC in accordance with the regulations and guidelines outlined in the Animal Welfare Act and the Guide for the Care and Use of Laboratory Animals, the recommendations of the Weatherall report [20], and the principles described in the National Research Council’s Guide for the Care and Use of Laboratory Animals. The University of Wisconsin–Madison Institutional Biosafety Committee approved this work under protocol number B00000764. See study approval section below for animal protocol details.

### 2.3. Care and Use of Macaques

All animals were housed in enclosures with required floor space and fed using a nutritional plan based on recommendations published by the National Research Council. Dams were fed a fixed formula, extruded dry diet with adequate carbohydrate, energy, fat, fiber, mineral, protein, and vitamin content. Macaque dry diets were supplemented with fruits, vegetables, and other edible objects (e.g., nuts, cereals, seed mixtures, yogurt, peanut butter, popcorn, marshmallows, etc.) to provide variety to the diet and to inspire species-specific behaviors such as foraging. When needed, infants were fed 5% dextrose for the first 24 h of life and liquid formula subsequently. To further promote psychological well-being, animals were provided with food enrichment, structural enrichment, and/or manipulanda. Environmental enrichment objects were selected to minimize chances of pathogen transmission from one animal to another and from animals to care staff. While on study, all animals were evaluated by trained animal care staff at least twice each day for signs of pain, distress, and illness by observing appetite, stool quality, activity level, and physical condition. Animals exhibiting abnormal presentation for any of these clinical parameters were provided appropriate care by attending veterinarians. Prior to all minor/brief experimental procedures, macaques were sedated using ketamine anesthesia and monitored regularly until fully recovered from anesthesia.

The female macaques were co-housed with a compatible male and observed daily for menses and breeding. Pregnancy was detected by ultrasound examination of the uterus at approximately 20–24 gestation days (gd) following the predicted day of ovulation. The gd was estimated (+/−2 days) based on the dam’s menstrual cycle, observation of copulation, and the greatest length of the fetus at initial ultrasound examination which was compared to normative growth data in this species [21]. For physical examinations, virus inoculations, ultrasound examinations, blood or swab collections, the dam was anesthetized with an intramuscular dose of ketamine (10 mg/kg). Blood samples from the femoral or saphenous vein were obtained using a vacutainer system or needle and syringe. Pregnant macaques were monitored daily prior to and after viral inoculation for any clinical signs of infection (e.g., diarrhea, inappetence, inactivity, fever, and/or atypical behaviors).

### 2.4. Inoculation and Monitoring

**Figure 1:**
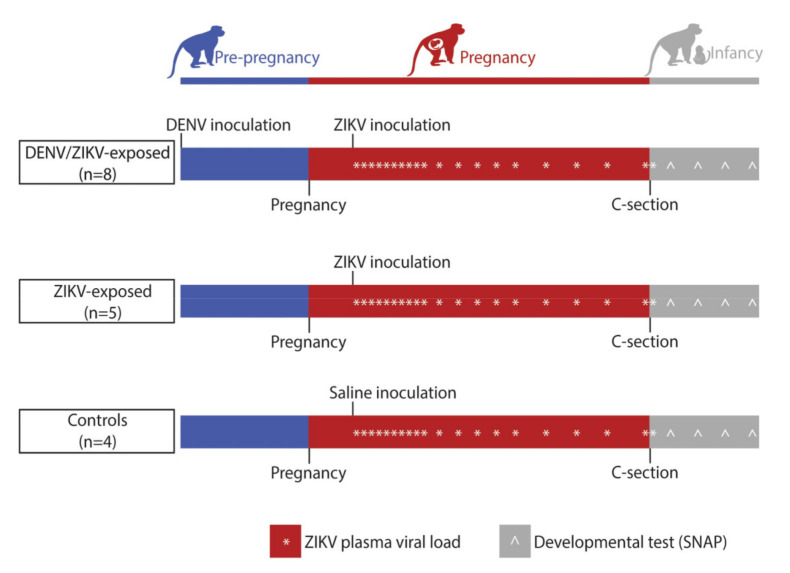
Experimental timeline schema. Female macaques (n = 8) were inoculated with DENV 1–2 months prior to breeding. Around gestational day 45, dams were either inoculated with ZIKV or saline. Blood was drawn daily to measure ZIKV plasma viral loads (*) for 10 days, then twice weekly until viremia cleared, then weekly until delivery by Cesarean section (C-section). Infants had blood drawn for a ZIKV plasma viral load within the first week of life and participated in weekly neurodevelopmental exams (Schneider Neonatal Assessment Protocol; SNAP) weekly (^) for the first month of life. Precise gestational days at inoculation and C-section are noted in Table 1, and average infant ages at developmental testing are noted in Supplementary Table S4.

Prior to pregnancy, eight macaques were inoculated with 1 × 10^4^ PFU DENV-2/US/BID-V594/2006 37–68 days prior to breeding (Table 1), as previously described [19]. Macaques were bred and, once pregnant, were inoculated subcutaneously with PBS or 1 × 10^4^ PFU Zika virus/*H.sapiens-tc/PUR/2015/PRVABC59_v3c2* (PRVABC59, GenBank: KU501215) over the cranial dorsum. This virus was originally isolated from a traveler to Puerto Rico and passaged three times on Vero cells (American Type Culture Collection (ATCC): CCL-81). The seed stock was obtained from Brandy Russell (CDC, Ft. Collins, CO). Virus stocks were prepared by inoculation onto a confluent monolayer of C6/36 cells (*Aedes albopictus* mosquito larval cells; ATCC: CCL-1660) with two rounds of amplification. Post-inoculation, the animals were closely monitored by veterinary and animal care staff for adverse reactions or any signs of disease. Blood was drawn for ZIKV qRT-PCR daily for 10 days following inoculation during pregnancy, then twice weekly until viremia cleared, then weekly until the end of pregnancy (Figure 1). Infants had blood drawn for ZIKV qRT-PCR immediately after delivery, or within the first week of life if the infant was born naturally (Figure 1).

### 2.5. Pregnancy Monitoring and Fetal Measurements

Weekly ultrasounds were conducted to observe the health of the fetus and to obtain measurements including fetal femur length (FL), biparietal diameter (BPD), head circumference (HC), and heart rate as previously described [22]. Growth curves were developed for FL, BPD, and HC using mean measurements and standard deviations from Tarantal et al. [21]. Ultrasound measurements were plotted against this normative data. Doppler ultrasounds to measure fetal heart rate were performed biweekly.

### 2.6. Cesarean Delivery

Infants were delivered by Cesarean section at approximately 160 gestational days (gd), about 6 days earlier than the average gestational age of a natural birth at the Wisconsin National Primate Center to ensure that the placenta could be collected for virologic and histologic evaluation. Amniotic fluid was collected just prior to infant delivery via aspiration with a syringe and needle inserted through the membranes into the amniotic fluid. Two animals were delivered by natural delivery (044-109 and 044-108) just prior to their scheduled Cesarean section; placenta could only be collected from 044-108, not 044-109.

### 2.7. Placental Pathology

Placentas were collected and sampled at the time of Cesarean section. Cotyledons were separated using sterile razor blades (separate blades for each disc) and were placed into separate sterile Petri dishes which were cooled on ice throughout the dissection. A 1/2”-wide center cut was made across the diameter of each cotyledon with a single-use razor blade and placed into a tissue cassette. Placental center cuts and cotyledon center cuts were fixed in 4% paraformaldehyde for 24 h, transferred to 70% ethanol, paraffin embedded, and sectioned for histology. Placental center cuts were evaluated via brightfield microscopy for pathologic lesions indicative of fetal vascular malperfusion, maternal vascular malperfusion, and inflammation involving the umbilical cord, chorionic plate, villous parenchyma, basal plate, decidua, and fetal membranes at the lateral margins of the placental discs. No significant pathologic changes were observed. Samples of chorionic plates and decidua were dissected from each cotyledon using sterile, single-use forceps. Cotyledon center cut sections were analyzed via brightfield microscopy for vasculopathies, infarctions, and chronic histiocytic intervillositis.

### 2.8. vRNA Isolation from Plasma and Tissues

RNA was extracted from 300 μL of plasma using the Viral Total Nucleic Acid Purification kit (Promega, Madison, WI, USA) on a Maxwell 48 RSC instrument. qRT-PCR was performed as previously described [23]. The limit of quantification for the assay is 100 copies/mL for qRT-PCR from plasma. Fetal and maternal–fetal interface tissues (placenta, decidua, umbilical cord, chorionic plate, fetal membranes) were preserved with RNAlater^®^ (Invitrogen, Carlsbad, CA, USA) at 4 °C for 24–72 h before the RNAlater was removed and the tissue was frozen at −80 °C. RNA was isolated from maternal and fetal tissues using a method described by Hansen et al. [24] and previously described in detail by Koenig M. et al. [17]. All tissue samples >0.1 copies/mg are considered positive for ZIKV vRNA. The fraction of ZIKV vRNA-positive cotyledons divided by the number of cotyledons assessed for viral loads were quantified to assess the distribution of vRNA throughout the placenta, decidua and chorionic plate.

### 2.9. Plaque Reduction Neutralization Test (PRNT)

Macaque serum samples from the DENV/ZIKV animals were screened for DENV neutralizing antibodies using a plaque reduction neutralization test (PRNT) immediately prior to ZIKV inoculation. Endpoint titrations of reactive sera, using a 90% cutoff (PRNT90), were performed against DENV-2 as previously described [25]. Briefly, DENV was mixed with 2-fold serial dilutions of serum for 1 h at 37 °C prior to being added to Vero cells, and neutralization curves were generated using GraphPad Prism version 9.0.0 for Windows (GraphPad Software, San Diego, CA, USA, www.graphpad.com. Accessed on 5 June 2021). The resulting data were analyzed by nonlinear regression to estimate the dilution of serum required to inhibit 90% of infection.

### 2.10. Infant Care

After delivery, infants were dried, stimulated, and received respiratory support as clinically indicated. Apgar scores were recorded at 1, 5, and 10 min of life using scoring criteria developed for rhesus macaques [26], except that temperature was not recorded in our scores. Infants were placed with their biological mothers upon recovery from anesthesia, or in the nursery until accepted by their biological or surrogate mother. Three of seventeen infants remained in the nursery for the entire duration of the study (30 days) due to rejection from their biological and/or surrogate mother as described in Supplementary Table S1. Infants who were nursery-reared were placed in peer groups at 29–30 days of life. Infants who remained in the nursery were fed 10% dextrose for the first day of life then formula ad lib. Once animals were housed with their biological or surrogate mothers, no supplemental formula was provided, except to 042-502, who required formula because of poor weight gain. Infants were removed from their cage for attempted pairings with surrogate adult females or neurodevelopmental assessments only.

### 2.11. Neurobehavioral Assessments

We evaluated neonatal macaque neurobehavior with a well-validated assessment of developed infants for rhesus macaques, termed the Schneider Neonatal Assessment for Primates (SNAP) [27,28,29,30,31], which is based on the human Brazelton Newborn Behavioral Assessment Scale [32]. Twenty-nine test items in the SNAP that aligned with the neurodevelopmental areas of interest and make up the Orientation, Motor maturity and activity, Sensory responsiveness, and State control developmental constructs [31,33] were scored with one-minute vocalizations contributing to both the Sensory and State control constructs (Supplementary Table S2). This neonatal neurobehavioral test has previously been used to define neonatal development of prenatally ZIKV-exposed infants [17]. The Orientation construct was further separated by sensory modality and task into subgroup scores to isolate specific deficit areas: visual orientation, visual tracking, auditory orientation, and focus (Supplementary Table S3). Ratings were based on a five-point Likert scale ranging from 0 to 2. The SNAP was administered between approximately 1:00–3:00 pm at 7, 14, 21, and 28 (+/−2) days of life (DOL), with the day of birth considered DOL 1 (Supplementary Table S4).

Infants were wrapped in a cloth towel and brought to a testing room with decreased sensory stimuli immediately following separation from their mother. Examiners were trained in standardized administration and scoring procedures by the SNAP developer, M. Schneider, requiring a check-out protocol prior to administration. Three examiners (M. Schneider, K. Ausderau, K. Bach) were present for all neurobehavioral testing and scoring to ensure test administration reliability (> 95%). Items were administered in a consistent sequence across all animals to optimize performance and decrease handling time. Assessments were hand-scored on a printout of the scoring form during administration. Forms were transferred to electronic versions by Qualtrics Survey Software (Qualtrics, Provo, UT, USA). Higher scores reflect optimal scores; variables in which higher numbers do not reflect optimal scores were reverse coded (Supplementary Table S2) with IBM SPSS software. Test items which represent repetitions of the same skill, such as right, left, up, and down orientation, were averaged together before calculating the average of all the test items within a construct, as described previously [31,34].

### 2.12. Statistical Analyses

#### 2.12.1. Neonatal Development

Linear mixed effect modeling with animal-specific random effects was used to compare development trajectories within increasing gestational age between experimental groups (control, ZIKV, DENV/ZIKV) for the following constructs: Orientation, Motor maturity, Sensory responsiveness, and State control. Model assumptions were verified by examining residual plots. The analyses were adjusted by the following baseline characteristics: sex, number of days in the nursery and birth weight. Growth trajectories were quantified by estimating the slope parameters which were reported along with the corresponding 95% confidence intervals. Post hoc pairwise comparisons between experimental groups were conducted using Tukey’s Honestly Significant Difference (HSD) method. All reported *p*-values are two-sided and a *p*-value of 0.05 was used to define statistical significance. Statistical analyses were conducted using SAS software (SAS Institute, Cary, NC, USA), version 9.4.

#### 2.12.2. Pregnancy and Infant Individual Characteristics

Viral parameters measured on a quantitative scale (peak viral load, viremia duration, etc.) were compared between groups using a nonparametric Wilcoxon rank sum test. Binary viral parameters (e.g., viremia duration greater than 21 days) were compared between groups using Fisher’s exact test.

Gestational age standardized growth parameters for fetal head circumference (HC), biparietal diameter (BPD), abdominal circumference, and femur length were evaluated by calculating gestational age-specific *z*-values from normative fetal growth parameters [21]. Specifically, polynomial regression analyses of the normative means and standard deviations on gestational age were conducted to calculate gestational age-specific *z*-values for HC, BPD, abdominal circumference, and femur length. The primary analyses were the comparisons of the control group vs. the ZIKV group and the control group vs. the DENV/ZIKV group. In order to maximize the sensitivity of the analyses in this exploratory study with a small sample size, no formal adjustment for multiple comparisons was made. Secondary analyses were comparisons between the control group vs. the combined ZIKV-exposed fetus group (ZIKV; DENV/ZIKV). Fetal head growth relative to femur length growth were evaluated by calculating the BPD/femur length and HC/femur length ratios. Since the ratios were non-normally distributed, log-transformed ratios were used before conducting comparisons between groups. Linear mixed effects modeling with animal-specific random effects was used to analyze the fetal growth trajectories with advancing gestational age. In these analyses, the fetal growth parameters were regressed on gestational age. An autoregressive correlation structure was used to account for correlations between repeated measurements of growth parameters over time. The growth trajectories were quantified by calculating the regression slope parameters which were reported along with the corresponding 95% confidence intervals (CI). Fetal growth was evaluated both within and between groups.

#### 2.12.3. Predictors of Neonatal Neurobehavior

Univariate and multivariate linear regression analyses were conducted using identify predictors for neonatal neurobehavior. A total of 9 maternal and infant factors were included in the initial non-parsimonious multivariate regression analysis. The fraction of vRNA-positive cotyledons was calculated by summing the total number of vRNA positive cotyledons in the placenta, decidua, chorionic plate, and dividing by the number of cotyledons assayed. Backward selection with a selection criterion of *p* < 0.10 (given a small sample size to maximize sensitivity in this exploratory study) was used for variable selection to identify a parsimonious model with each outcome variable. A sensitivity analysis for the variable selection was conducted by applying forward selection variable selection for each outcome variable. Model assumptions were verified by examining residual plots. Multicollinearity was examined by evaluating variance inflation factors for each predictor of the final parsimonious regression model. Model fit was quantified by calculating the adjusted (by number of covariates) R2 values.

## 3. Results

### 3.1. Neonatal Development

**Figure 2:**
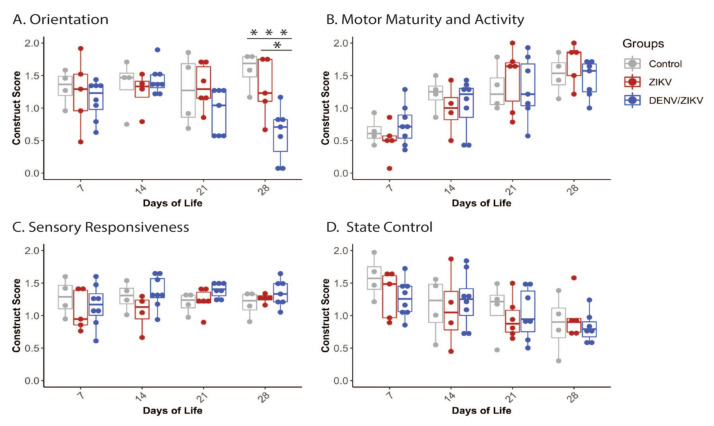
Neonatal neurodevelopment in the first month of life. Neurodevelopment was measured by the SNAP at 7, 14, 21, and 28 days of life. Scores in the (**A**) Orientation, (**B**) Motor Maturity and Activity, (**C**) Sensory Responsiveness, and (**D**) State control constructs are illustrated. * = *p* < 0.05, *** = *p* < 0.001.

We hypothesized that infant macaques exposed to ZIKV prenatally would have significant deficits that are apparent in the neonatal period, and that infants born to dams with DENV immunity would have more significant deficits, using a standardized macaque assessment designed to define development in the first four weeks of life [33,35,36,37]. Infants in all three groups (control, ZIKV, and DENV/ZIKV) scored similarly in the Orientation construct at the first two testing time points (7 and 14 DOL) (Figure 2A). Infants in the DENV/ZIKV group scored lower on orientation variables overall, with a distinct decline beginning at day 21. By day 28, the DENV/ZIKV-exposed infants scored significantly lower on orientation variables when compared to control infants (*p* < 0.01) and ZIKV-exposed infants (*p* < 0.05). While statistically significant, orientation development at 28 days of life is heterogeneous for both the ZIKV-exposed and DENV/ZIKV-exposed infants. Three of five of the ZIKV-exposed infants and all of the DENV/ZIKV-exposed infants have lower scores than the median control infants. When the ZIKV-exposed infant group and DENV/ZIKV infant group were combined, a significant difference between all ZIKV-exposed infants and control infants was still observed (*p* = 0.0047), even though there was no overall difference between the controls and ZIKV-exposed infant groups when compared separately.

Motor activity and maturity scores increased across testing days for all groups, as would be expected through maturation (Figure 2B). Sensory responsiveness scores stay relatively stable across all groups with a slight increase noted in both the ZIKV and DENV/ZIKV groups (Figure 2C). State control decreased across days of testing for all groups, as expected due to increased alertness and resistance to human-facilitated testing conditions (Figure 2D). There were no significant differences in Motor maturity and activity, Sensory responsiveness, or State control scores at any time point across groups (ZIKV vs. control, DENV/ZIKV vs. control, ZIKV vs. DENV/ZIKV) (Supplementary Tables S5 and S6).

**Figure 3:**
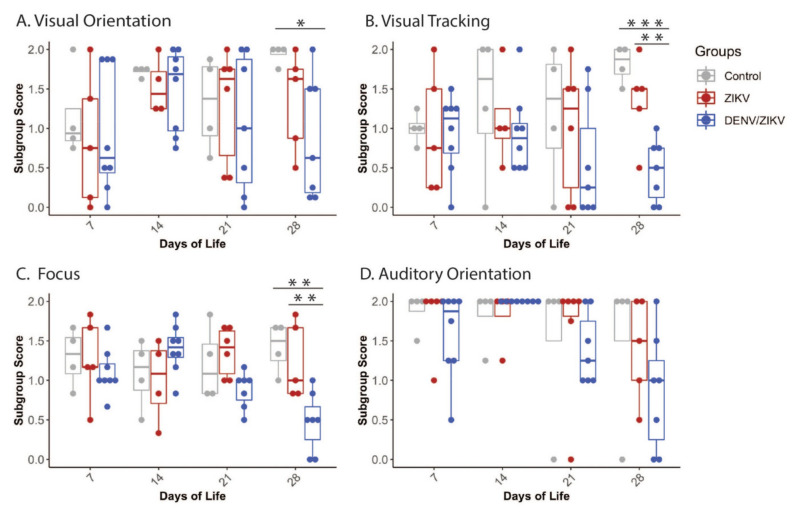
Neonatal neurodevelopment in the orientation domain. Orientation construct was separated into a subgroup analysis of (**A**) Visual orientation, (**B**) Visual tracking, (**C**) Focus, and (**D**) Auditory orientation. * = *p* < 0.05, ** = *p* < 0.01, *** = *p* < 0.001.

The Orientation construct was further separated by sensory modality and task into subgroups to isolate specific deficit areas. These Orientation subgroups are independent measures because they are measured through different tasks, but overall remain related to the broad category of orientation. DENV/ZIKV-exposed infants scored lower than controls in the subgroups of Visual orientation, Visual tracking and Focus, and had lower scores than ZIKV-exposed infants in the Focus subgroup (Figure 3, Supplementary Tables S5 and S6). Orientation subgroup differences persisted when the ZIKV and DENV/ZIKV infant groups were combined and compared with control infants in all three Orientation subgroups pertaining to vision (i.e., Visual orientation, Visual tracking and Focus) (Supplementary Table S6). ZIKV-exposed infants may have differences in orientation, although these were not significant compared to the DENV/ZIKV infants, potentially suggesting a continuum of deficits in these areas.

### 3.2. Maternal ZIKV Infection Characteristics

**Figure 4:**
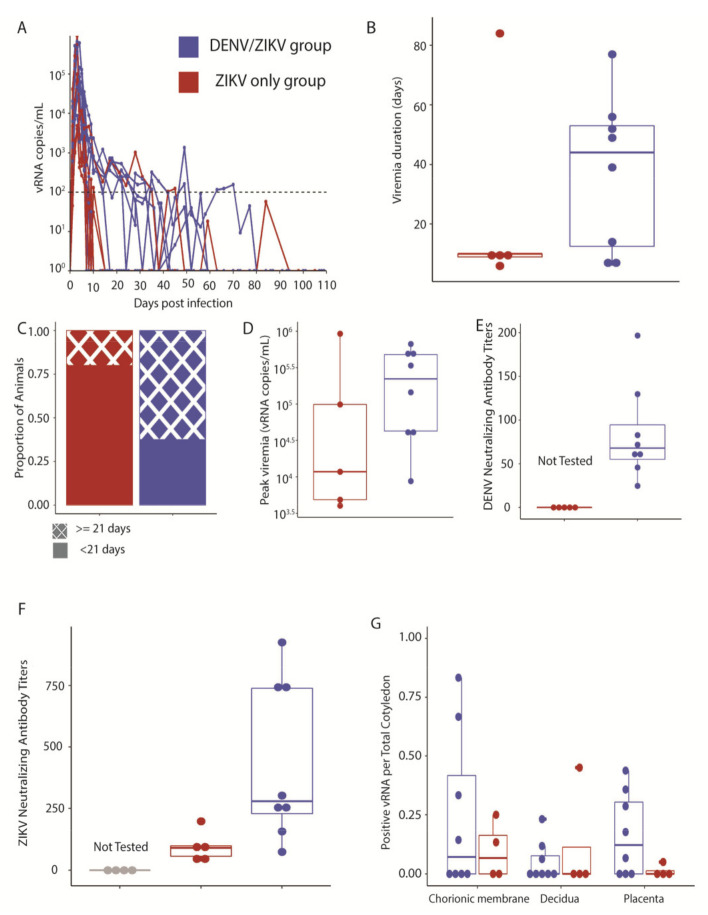
Maternal ZIKV infection characteristics and neutralizing antibody responses. (**A**) Plasma viremia curves for individual animals, denoted by different lines for each animal. Dashed line is the limit of quantification. (**B**) Duration of viremia, from the day of inoculation until the last day of a positive plasma viral load (*t*-test, *p*-value = 0.4638). (**C**) Proportion of dams within each group with viremia duration greater or less than 21 days (*t*-test, *p*-value = 0.2657). (**D**) Peak viral plasma loads, defined as the highest plasma vRNA titer (*t*-test, *p*-value = 0.4208). (**E**) DENV PRNT90 on the day of ZIKV inoculation (ZIKV-only animals not tested). (**F**) ZIKV PRNT90 at 28 days post-ZIKV inoculation (control animals not tested). (**G**) ZIKV vRNA distribution within the maternal–fetal interface, displayed as the proportion of cotyledons with a positive viral load.

We evaluated whether maternal ZIKV infection characteristics differed between pregnant macaques with and without history of prior DENV infection by closely measuring maternal viral loads after ZIKV infection and assessing vRNA distribution in the maternal–fetal interface. All ZIKV-challenged dams demonstrated plasma viremia starting on day 1 post-inoculation and reached peak viral loads 2 to 5 days post-infection (Figure 4A). Viremia duration did not differ significantly between ZIKV-inoculated dams with and without a history of DENV infection 1–2 months prior to pregnancy (Figure 4B). Viremia duration also did not differ between dams with and without DENV immunity when it was considered as a discrete variable, either greater or less than 21 days (Figure 4C). Peak plasma viral loads did not significantly differ between dams with and without DENV immunity (Figure 4D). DENV-neutralizing antibodies were present in all DENV/ZIKV dams on the day of ZIKV inoculation (Figure 4E). All ZIKV-inoculated dams developed ZIKV neutralizing antibodies (Figure 4F). Maternal–fetal interface ZIKV vRNA distribution, or fraction of positive viral loads within the different layers of the cotyledon, i.e., placenta, decidua, and chorionic plate, did not differ between ZIKV-exposed dams with and without DENV immunity (Figure 4G, Supplementary Tables S7–S9). Overall, maternal viremia characteristics and maternal–fetal interface viral distribution were similar in dams inoculated with ZIKV, regardless of their history of prior DENV infection.

### 3.3. Fetal Growth, Placental Pathology and Infant Health

**Figure 5:**
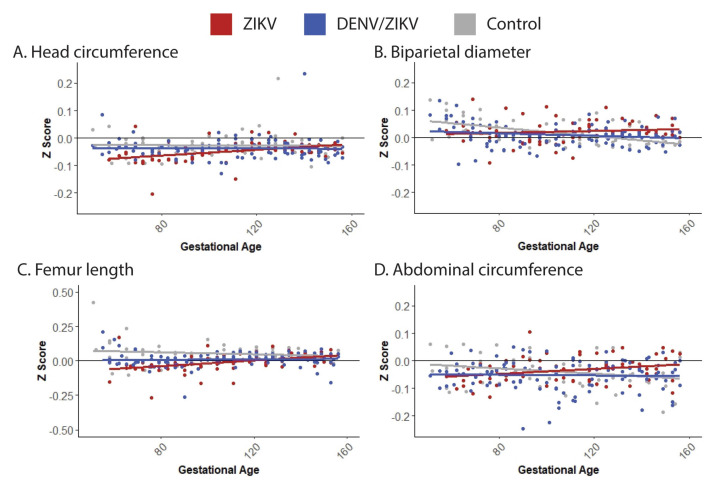
Fetal growth parameter trajectories for ZIKV-exposed, DENV/ZIKV-exposed and control fetuses. Fetal growth parameters throughout gestation were calculated as Z scores relative to age-matched fetal macaque parameters [21] and graphically represented as a linear trajectory. Fetal head circumference growth trajectory is represented in (**A**), biparietal diameter in (**B**), femur length in (**C**), and abdominal circumference in (**D**).

We sought to determine what differences existed between ZIKV-exposed pregnancies with and without DENV immunity in regard to fetal growth trajectory, placental pathology, and infant health. We were especially interested in fetal head circumferences and biparietal diameters because head circumferences are disproportionately affected in ZIKV-exposed human fetuses [38]. We did not identify a slower growth trajectory in either of the ZIKV-exposed groups in any of the fetal measurements (Figure 5), even when they were corrected for femur length to take into account asymmetric growth of the head and femur (Supplementary Tables S10 and S11). Placental pathology, as defined by the presence or absence of chronic histiocytic intervillositis, villous stromal calcifications, and vasculopathies, was also not significantly different between either of the ZIKV-exposed groups or controls (Table 2, Supplementary Tables S9 and S12). In summary, we did not identify significant differences in placental parameters between ZIKV-infected dams with and without DENV immunity.

We hypothesized that DENV/ZIKV-exposed infants may have a more difficult time gaining a healthy amount of weight through their first month of life. We did not find any significant differences in Apgar scores, a score which assesses the neonatal transition to life outside the uterus, at 1, 5, or 10 min of life between ZIKV-exposed neonates with and without DENV immunity and control neonates (Supplementary Tables S9 and S13) even when the ZIKV-exposed neonates were grouped together to increase sample size. We also did not identify differences in the weight gain trajectory during the first month of life between the ZIKV-only, DENV/ZIKV, and control groups (Supplementary Tables S8 and S14). Infants were assessed for ZIKV vRNA in plasma and urine, and these were negative (Supplementary Table S15), as has been found in other studies of congenital ZIKV exposure [17]. Overall, ZIKV-exposed infants with and without DENV immunity had similar infant health characteristics as control infants.

### 3.4. Predictors of Neonatal Neurodevelopment

We hypothesized that multiple factors may influence developmental outcomes because individual pregnancy and infant factors may provide an overly simplistic approach to identifying factors that predict development. Therefore, we explored how key maternal ZIKV infection and infant health factors influenced developmental outcomes of all infants (control, ZIKV, DENV/ZIKV) in multivariate regression analysis. We selected nine maternal and infant factors that we hypothesized would be most impactful. We were constrained by model restrictions, which required use of only continuous variables: maternal viremia characteristics, viral distribution in the MFI, placental weight, birth weight, and maternal DENV-neutralizing antibody titer (PRNT90). Our outcomes were SNAP scores at 28 days of life, because that is where we observed the most heterogeneity among infants. Individual factors were significantly associated with developmental outcome scores in a univariate regression analysis in only a few cases, with fetal head growth significantly associated with Visual Orientation scores, maternal plasma viremia and peak plasma viral load associated with State control construct scores, and placental weight associated with Motor maturity and activity construct scores (Table 3).

Seeking to explore how these factors together influenced developmental outcomes, we performed a multivariate regression analysis and found that specific combinations of maternal ZIKV infection and fetal health markers were significantly associated with the Orientation construct score (R2 = 0.85), the Motor maturity and activity construct score (R2 = 0.52), and the State control construct score (R2 = 0.19) (Table 3). Next, we defined the combination of predictors associated with subgroups of the Orientation construct, because this is the developmental area where we observed significant differences between groups. Multiple maternal and fetal factors were associated with the Visual orientation subgroup (R2 = 0.96), Attention subgroup (R2 = 0.76), and Auditory orientation subgroup (R2 = 0.94). Maternal viremia duration appears to be a common factor influencing most developmental outcomes because it was associated in five of the six multivariate models. Furthermore, a shorter viremia duration appears to be associated with higher SNAP scores, because the standardized regression coefficient was negative in all the models. A maternal history of DENV infection influenced developmental outcomes the least, as only one of six multivariate models identified this factor as a significant predictor of developmental outcomes when combined with the other pregnancy and infant factors.

## 4. Discussion

We found that maternal DENV exacerbates orientation developmental deficits in ZIKV-exposed infant macaques, specifically in the tasks of visual tracking, focus, and visual orientation. The presence of DENV immunity alone did not predict developmental outcomes, but together with specific maternal ZIKV infection characteristics and pregnancy parameters, it significantly predicted developmental outcomes. This is the first time that the influence of prior maternal DENV infection on ZIKV-exposed infant development has been defined and specific predictors of infant neurodevelopment in a controlled preclinical model have been identified.

We identified a specific pattern of orientation deficits in the DENV/ZIKV-exposed infants, whereby DENV/ZIKV-exposed infants had the lowest scores, ZIKV-only exposed infants scored in the middle range and control infants had the highest (optimal) scores. Our finding of neonatal orientation deficits may have cascading implications for cognitive skills outside of infancy. Orientation deficits in the neonatal period predict learning and memory deficits during adolescence in a preclinical macaque model of prenatal alcohol and stress exposure [39]. Long-term developmental studies of these infant macaques are underway to understand the developmental trajectory and understand how visual orientation deficits in the first month of life may impact future neurodevelopment. These long-term studies may also uncover deficits, such as motor, social cognition, language and visual reception deficiencies, which would match the unique deficits seen in human ZIKV-exposed children during childhood [1,2,40]. We specifically focused on the neonatal age range for this study because identification of deficits in early infancy provides the opportunity to intervene with early intervention therapies that improve functional outcomes. In addition, the current neurodevelopmental testing approach was validated in infant macaques less than a month old and has been useful in predicting later outcomes [27,28,29,31,34]. Early infant orientation deficits may provide a lens into subtle but important early delays that predict significant deficits in language and motor development that are commonly observed in human children. Most significantly, by identifying the early developmental limitations, interventions that focus on the unique deficits will maximize functional outcomes and prevent cascading effects that spread into other developmental domains.

Maternal ZIKV plasma viremia duration was the most consistent predictor of all the developmental outcomes in our multivariate model. Maternal ZIKV plasma viremia duration, in combination with other pregnancy factors, significantly predicted neonatal development scores in five of the six significant developmental outcomes. Most importantly, there was a negative relationship between maternal ZIKV viremia duration and higher scores in developmental outcomes, which means that a shorter viremia duration is associated with better developmental outcomes. There were no other maternal ZIKV infection characteristics or pregnancy parameters that predicted developmental outcomes as consistently as maternal ZIKV viremia duration did. Our finding that shorter maternal viremia duration is associated with better development extends what is known from human studies, which is that shorter maternal viremia duration is associated with lower rates of fetal demise and infant cerebral abnormalities [41]. Thus, shortening maternal viremia duration improves pregnancy outcomes and infant developmental outcomes in human and preclinical model studies, respectively, and should be considered a target for new ZIKV antivirals.

We selected the neutralizing DENV antibody titer as a predictor because it is considered the gold standard of quantifying circulating neutralizing DENV antibodies [42]; however, DENV-neutralizing antibody titer was the least important parameter in predicting developmental outcomes. Maternal DENV immunity was not important in predicting the majority of developmental outcomes in a multivariate analysis even though DENV/ZIKV-exposed infants had lower orientation developmental scores. This suggests that the role of maternal DENV immunity on neurodevelopmental outcomes of ZIKV-exposed infants is complex and different maternal DENV immunity variables may be more important. Other features of the DENV antibody response, such as cross-reactivity with ZIKV or specific DENV antibodies binding cross-reactive epitopes (as in the iELISA; [43]), may be more important and should be evaluated in future studies. Our study provides the first clue that pre-existing maternal DENV immunity may worsen developmental deficits in ZIKV-exposed infants, and that it must be considered alongside maternal ZIKV infection characteristics and pregnancy parameters in influencing neurodevelopment. Long-term developmental studies in humans need to define how maternal DENV immunity may influence the heterogeneity of developmental deficits in ZIKV-exposed children. While current studies suggest that approximately 30% of typically developing human infants have late-manifesting deficits [1,2], they have yet to examine the potential role that DENV immunity may contribute to the spectrum of deficits observed.

In conclusion, the role that maternal DENV immunity plays in influencing long-term neurodevelopmental outcomes in human infants should be evaluated, as these infant macaques had worse outcomes than ZIKV-exposed infants. Because the DENV-neutralizing antibody titer was not the most important predictor of development in our study, other DENV-related variables, such as DENV iELISA titers and cellular immunity parameters, should be utilized in human developmental studies. In addition, novel ZIKV antivirals that shorten maternal ZIKV duration may result in improved infant neurodevelopment, and this endpoint should be evaluated in future ZIKV antiviral studies. Our study begins to identify early developmental deficits that may have cascading effects on long-term outcomes, including learning and memory skills, and will only be identified in the years to come as children born during the 2015–2016 ZIKV pandemic grow older. Our finding that a history of maternal DENV infection worsens neonatal development should shape how we screen women during pregnancy to target their children for early surveillance and developmental evaluation. Early developmental predictors specifically related to orientation skills will provide an opportunity for targeted early intervention in human infants and children to support remediation and achievement of developmental milestones.

## Figures and Tables

**Table 1:** Maternal medical and inoculation history.

Group	Dam ID	Inoculation Prior to Pregnancy	Inoculation during Pregnancy	Gestational Day at Inoculation	Dam Age at Inoculation during Pregnancy (Years)	Gestational Day at Infant Delivery	Number of Previous Pregnancies	Pregnancy Data Presented in Previous Publication
Control	044-105	-	PBS	42	11	161	3	-
	044-106	-	PBS	48	15	160	6	-
	044-107	-	PBS	48	8	163	5	-
	044-108	-	PBS	48	12	159	5	-
ZIKV	044-101	DMEM + FBS	ZIKV	48	16	160	6	(18)
	044-102	DMEM + FBS	ZIKV	45	17	160	7	(18)
	044-103	DMEM + FBS	ZIKV	45	13	159	3	(18)
	044-104	DMEM + FBS #	ZIKV	45	7	159	2	(18)
	044-109	-	ZIKV	48	16	160	7	-
DENV/ZIKV	042-102	DENV	ZIKV	46	7	160	2	(18)
	042-104	DENV	ZIKV	46	12	160	5	(18)
	042-101	DENV	ZIKV	48	13	160	5	(18)
	042-103	DENV	ZIKV	45	6	159	1	(18)
	042-107	DENV	ZIKV	48	11	160	3	(18)
	042-105	DENV	ZIKV	49	15	161	6	(18)
	042-108	DENV	ZIKV	45	8	161	3	(18)

# Dulbecco’s Modified Eagle’s Medium (DMEM), fetal bovine serum (FBS).

**Table 2:** Placental pathology.

Group	Dam	Infarctions/ Total Cotyledons	Chronic Histiocytic Intervillositis	Villous Stromal Calcifications	Vasculopathy	Total Placental Weight (g)
Control	044-105	1/17	Absent	Present	Absent	111.08
	044-106	1/8	Absent	Present	Absent	106.5
	044-107	0/9	Absent	Present	Present	144.48
	044-108	5/11	Absent	Present	Absent	122.92
ZIKV	044-101	5/20	Absent	Present	Absent	172.59
	044-102	6/15	Absent	Present	Absent	123.87
	044-103	0/16	Absent	Absent	Absent	134.49
	044-104	2/11	Absent	Absent	Absent	120.48
DENV/ZIKV	042-101	3/14	Absent	Present	Absent	104.4
	042-102	1/13	Present	Present	Absent	111.9
	042-103	0/17	Absent	Present	Absent	120.06
	042-104	4/15	Absent	Present	Absent	95.39
	042-105	4/16	Absent	Absent	Absent	119.97
	042-106	8/15	Absent	Present	Present	120.14
	042-107	4/14	Absent	Present	Absent	139.74
	042-108	5/14	Absent	Present	Absent	129.54

**Table 3:** Multivariate analysis of maternal ZIKV infection characteristics and pregnancy parameters.

Outcomes	Predictors	Univariate Regression Analysis		Multivariate Regression Analysis			
		Correlation Coefficient	*p*-Value	Standardized Regression Coefficient	*p*-Value	Variance Inflation Factor	R2 Final Multivariate Model
Orientation construct	Duration of maternal plasma viremia	0.10	0.7651	−0.92	0.0112	3.80	0.85
	Peak maternal plasma viral load	−0.21	0.5193				
	Fraction of vRNA -positive cotyledons	−0.21	0.4479	0.72	0.0197	3.10	
	DENV immunity (PRNT90)	0.08	0.7654				
	Infarctions/total cotyledons	−0.09	0.7603	0.49	0.0153	1.30	
	Fetal head circumference trajectory	0.01	0.9827	0.47	0.0169	1.20	
	Fetal biparietal diameter growth trajectory	−0.39	0.1331				
	Total placental weight	0.29	0.2974	1.08	0.0008	1.50	
	Birth weight	0.14	0.6117				
Focus subgroup of Orientation construct	Duration of maternal plasma viremia	0.01	0.9736	−1.07	0.0171	3.8	0.76
	Peak maternal plasma viral load	−0.21	0.5198				
	Fraction of vRNA -positive cotyledons	−0.26	0.3471	0.84	0.029	3.1	
	DENV immunity (PRNT90)	0.07	0.7967				
	Infarctions/total cotyledons	−0.07	0.8174	0.49	0.0395	1.3	
	Fetal head circumference trajectory	0.06	0.8223	0.5	0.0342	1.2	
	Fetal biparietal diameter growth trajectory	−0.38	0.1417				
	Total placental weight	0.31	0.2591	1.04	0.003	1.5	
	Birth weight	0.04	0.8821				
Visual Orientation subgroup of Orientation construct	Duration of maternal plasma viremia	−0.1	0.7684	−0.63	0.0003	7.90	0.98
	Peak maternal plasma viral load	0.05	0.8793				
	Fraction of vRNA -positive cotyledons	−0.40	0.1428	0.30	0.0018	4.60	
	DENV immunity (PRNT90)	−0.03	0.9124	0.44	0.0001	2.10	
	Infarctions/total cotyledons	0.18	0.5189	0.17	0.0078	3.40	
	Fetal head circumference trajectory	−0.53	0.0331 *				
	Fetal biparietal diameter growth trajectory	−0.78	0.0004 *	−0.77	<0.0001	3.40	
	Total placental weight	0.48	0.0729				
	Birth weight	0.13	0.6220	0.57	0.0002	4.80	
Visual tracking subgroup of Orientation construct	Duration of maternal plasma viremia	0.12	0.7141	No significant predictors			
	Peak maternal plasma viral load	−0.06	0.8449				
	Fraction of vRNA -positive cotyledons	−0.11	0.6921				
	DENV immunity (PRNT90)	−0.05	0.8572				
	Infarctions/total cotyledons	−0.29	0.2990				
	Fetal head circumference trajectory	−0.15	0.5875				
	Fetal biparietal diameter growth trajectory	−0.4	0.1244				
	Total placental weight	0.44	0.0971				
	Birth weight	0.19	0.4732				
Auditory orientation subgroup of Orientation construct	Duration of maternal plasma viremia	0.21	0.5101	−1.8	0.0011	8.1	0.94
	Peak maternal plasma viral load	−0.32	0.3148	−0.77	0.0045	3.2	
	Fraction of vRNA -positive cotyledons	−0.05	0.8646	1.88	0.0008	7.4	
	DENV immunity (PRNT90)	0.17	0.5205				
	Infarctions/total cotyledons	0.07	0.8151				
	Fetal head circumference trajectory	0.32	0.2323	0.57	0.0066	2.2	
	Fetal biparietal diameter growth trajectory	−0.07	0.8054	−0.94	0.0012	2.3	
	Total placental weight	0.05	0.8643				
	Birth weight	0.04	0.8884	1.27	0.0011	4	
Motor maturity & activity construct	Duration of maternal plasma viremia	0.57	0.0541				0.52
	Peak maternal plasma viral load	0.40	0.1925	0.56	0.0577	1.2	
	Fraction of vRNA -positive cotyledons	0.43	0.1084				
	DENV immunity (PRNT90)	0.26	0.3248				
	Infarctions/total cotyledons	−0.14	0.6248	−0.75	0.0776	2.6	
	Fetal head circumference trajectory	−0.01	0.9567	0.83	0.0332	1.9	
	Fetal biparietal diameter growth trajectory	−0.12	0.6707	−1.08	0.0458	3.9	
	Total placental weight	0.62	0.0128 *				
	Birth weight	0.30	0.2648				
Sensory responsiveness construct	Duration of maternal plasma viremia	−0.24	0.4620	No significant predictors			
	Peak maternal plasma viral load	0.19	0.5567				
	Fraction of vRNA -positive cotyledons	−0.1	0.7102				
	DENV immunity (PRNT90)	−0.02	0.9483				
	Infarctions/total cotyledons	0.21	0.4580				
	Fetal head circumference trajectory	−0.44	0.0872				
	Fetal biparietal diameter growth trajectory	−0.05	0.8541				
	Total placental weight	−0.08	0.7905				
	Birth weight	0.02	0.9309				
State control construct	Duration of maternal plasma viremia	−0.66	0.0187 *	−0.51	0.0877	1.00	0.19
	Peak maternal plasma viral load	−0.63	0.0283 *				
	Fraction of vRNA -positive cotyledons	−0.41	0.1240				
	DENV immunity (PRNT90)	−0.24	0.3743				
	Infarctions/total cotyledons	−0.05	0.8693				
	Fetal head circumference trajectory	−0.01	0.9828				
	Fetal biparietal diameter growth trajectory	−0.07	0.8034				
	Total placental weight	−0.28	0.3147				
	Birth weight	−0.07	0.8029				

* *p*-value < 0.05 in univariate regression analysis.

## Data Availability

Data are available: https://go.wisc.edu/6g9502.

## Supplementary Materials

The following are available online at https://www.mdpi.com/article/10.3390/v13091878/s1, Table S1. Infant demographics and social groups, Table S2. Test items included in each SNAP construct, Table S3. Test items included in SNAP Orientation construct subgroups, Table S4. Infant age at SNAP administration, Table S5. SNAP Construct and Orientation construct subgroup descriptive statistics, Table S6. SNAP construct and Orientation construct subgroup comparisons, Table S7. Maternal–fetal interface tissue viral loads, Table S8. Maternal ZIKV infection characteristics descriptive statistics, Table S9. Dam and infant ZIKV infection and health parameter group comparisons, Table S10. Fetal growth parameter trajectories, Table S11. Fetal growth trajectory group comparisons, Table S12. Maternal–fetal interface tissue descriptive statistics, Table S13. Apgar score descriptive statistics, Table S14. Infant weight gain descriptive statistics, Table S15. Neonatal body fluid viral loads.
