# Supplementary material for: Neonatal Development in Prenatally Zika Virus-Exposed Infant Macaques with Dengue Immunity
Source: Viruses. 2021 Sep 20;13(9):1878. doi: 10.3390/v13091878 (PMC8473338; doi:10.3390/v13091878)
Supplement: Supplementary file 1 [file viruses-13-01878-s001.zip › viruses-1291339-supplementary.pdf]

Supplemental Information

Supplemental Table S1. Infant demographics and social

Infant ID	Group	Sex	Days in nursery (alone or with peer)	Final social group	Final social group explanation
044-507	Control	Female	6 days	Mother (surrogate)	Biological mother rejected infant
044-505	Control	Female	4 days	Mother (surrogate)	Biological mother rejected infant
044-506	Control	Female	29 days	Nursery (peer group)	Biological mother and surrogate mother rejected infant. Placed in peer group with colony infants.
044-508	Control	Female	0 days	Mother (biological)	
044-504	ZIKV	Female	1 day	Mother (surrogate)	Biological mother rejected infant
044-503	ZIKV	Male	5 days	Mother (biological)	
044-502	ZIKV	Male	27 days	Nursery (peer group)	Biological mother rejected the infant and no surrogates available.
044-501	ZIKV	Male	1 day	Mother (biological)	
044-509	ZIKV	Male	0 days	Mother (biological)	
042-502	DENV/ZIKV	Female	5 days	Mother (surrogate)	Biological mother rejected infant
042-504	DENV/ZIKV	Female	1 day	Mother (biological)	
042-501	DENV/ZIKV	Male	0 days	Mother (biological)	
042-503	DENV/ZIKV	Female	15 days	Mother (surrogate)	Biological mother rejected infant. Placed in nursery before placement with surrogate at 15 days of age. Deceased at 17 days of age
042-507	DENV/ZIKV	Female	1 day	Mother (biological)	
042-505	DENV/ZIKV	Female	0 days	Mother (biological)	
042-508	DENV/ZIKV	Male	27 days	Nursery (peer group)	Biological mother rejected infant and no surrogates available.
042-506	DENV/ZIKV	Female	13 days	Mother (surrogate)	Biological mother rejected infant

Supplemental Table S2. Test items included in each SNAP construct

Orientation	Motor Maturity & Activity	Sensory	State Control
Visual orientation	Active power	Calming self*	Irritability
Visual tracking	Passive*	Rotation test	Calming self*
Duration of looking	Coordination	Parachute	Struggle during test*
Attention	Response speed	One-minute vocalization**	Predominant state*
Distractible*	Motor activity	Galants*	One-minute vocalizations**
Auditory Orientation	Maintenance balance	Head posture prone	Response intensity*
	Spontaneous crawl	Tactile response	Soothability*
			Consolability
			Tremulousness*

			Inversion*

*reverse coded

**vocalizations were converted into a number using number of vocalizations per minute

Supplemental Table S3. Test items included in SNAP Orientation construct subgroups

Visual orientation	Visual tracking	Auditory orientation	Focus
Visual orientation- left	Visual follow- horizontal	Auditory Orientation- right	Attention
Visual orientation- right	Visual follow- vertical	Auditory Orientation- left	Distractible*
Visual orientation- up			Duration of looking
Visual orientation- down			

*reverse coded

Supplemental Table S4. Infant age at SNAP administration

	Control		ZIKV		DENV/ZIKV		p-value
	Mean age (days)	SD	Mean age (days)	SD	Mean age (days)	SD	
Age at SNAP #1	6.75	0.96	7.00	1.22	6.88	1.13	0.95
Age at SNAP #2	14.00	1.15	14.80	1.30	13.88	1.13	0.40
Age at SNAP #3	21.50	1.00	21.60	0.55	20.57	0.98	0.12
Age at SNAP #4	28.00	0.82	28.60	0.89	27.57	0.98	0.20

Standard deviation (SD)

Supplemental Table S5. SNAP Construct and Orientation construct subgroup descriptive statistics

Construct/Orientation construct subgroup	Week of life	Group	Median
Orientation	1	Control	1.36
		ZIKV	1.19
		DENV/ZIKV	1.17
		ZIKV & DENV/ZIKV	1.18
Orientation	2	Control	1.40
		ZIKV	1.19
		DENV/ZIKV	1.47
		ZIKV & DENV/ZIKV	1.33
Orientation	3	Control	1.32
		ZIKV	1.33
		DENV/ZIKV	1.00
		ZIKV & DENV/ZIKV	1.17
Orientation	4	Control	1.63
		ZIKV	1.26
		DENV/ZIKV	0.66
		ZIKV & DENV/ZIKV	0.96
Motor Maturity and Activity	1	Control	0.64
		ZIKV	0.49
		DENV/ZIKV	0.73
		ZIKV & DENV/ZIKV	0.61

Motor Maturity and Activity	2	Control	1.21
		ZIKV	1.11
		DENV/ZIKV	1.04
		ZIKV & DENV/ZIKV	1.08
Motor Maturity and Activity	3	Control	1.30
		ZIKV	1.41
		DENV/ZIKV	1.26
		ZIKV & DENV/ZIKV	1.34
Motor Maturity and Activity	4	Control	1.52
		ZIKV	1.68
		DENV/ZIKV	1.40
		ZIKV & DENV/ZIKV	1.54
Sensory Responsiveness	1	Control	1.21
		ZIKV	1.09
		DENV/ZIKV	1.16
		ZIKV & DENV/ZIKV	1.13
Sensory Responsiveness	2	Control	1.28
		ZIKV	1.12
		DENV/ZIKV	1.28
		ZIKV & DENV/ZIKV	1.20
Sensory Responsiveness	3	Control	1.20
		ZIKV	1.28
		DENV/ZIKV	1.35
		ZIKV & DENV/ZIKV	1.32
Sensory Responsiveness	4	Control	1.30
		ZIKV	1.43
		DENV/ZIKV	1.34
		ZIKV & DENV/ZIKV	1.39
State Control	1	Control	1.55
		ZIKV	1.34
		DENV/ZIKV	1.28
		ZIKV & DENV/ZIKV	1.31
State Control	2	Control	1.11
		ZIKV	1.09
		DENV/ZIKV	1.26
		ZIKV & DENV/ZIKV	1.17
State Control	3	Control	1.07
		ZIKV	1.00
		DENV/ZIKV	1.10
		ZIKV & DENV/ZIKV	1.05
State Control	4	Control	0.86
		ZIKV	1.01
		DENV/ZIKV	0.89
		ZIKV & DENV/ZIKV	0.95
Orientation construct subgroup: Focus	4	Control	1.4
		ZIKV	1.2
		DENV/ZIKV	0.5
		ZIKV & DENV/ZIKV	0.8
Orientation construct subgroup: Visual Orientation	4	Control	1.9
		ZIKV	1.4
		DENV/ZIKV	0.9
		ZIKV & DENV/ZIKV	1.1
Orientation construct subgroup: Visual Tracking	4	Control	1.8
		ZIKV	1.4
		DENV/ZIKV	0.5
		ZIKV & DENV/ZIKV	0.8
Orientation construct subgroup: Auditory Orientation	4	Control	1.5
		ZIKV	1.4
		DENV/ZIKV	0.9
		ZIKV & DENV/ZIKV	1.1

Supplemental Table S6. SNAP construct and Orientation construct subgroup comparisons

Construct/Orientation construct subgroup	Week of life	Group	p-value
Orientation	1	Ctrl vs ZIKV	0.8734
		Ctrl vs DENV/ZIKV	0.676
		ZIKV vs DENV/ZIKV	0.9723
		Ctrl vs ZIKV&DENV/ZIKV	0.3555
Orientation	2	Ctrl vs ZIKV	0.8046
		Ctrl vs DENV/ZIKV	0.9557
		ZIKV vs DENV/ZIKV	0.5519
		Ctrl vs ZIKV&DENV/ZIKV	0.7552
Orientation	3	Ctrl vs ZIKV	0.9914
		Ctrl vs DENV/ZIKV	0.3843
		ZIKV vs DENV/ZIKV	0.3095
		Ctrl vs ZIKV&DENV/ZIKV	0.3663
Orientation	4	Ctrl vs ZIKV	0.4795
		Ctrl vs DENV/ZIKV	0.0008
		ZIKV vs DENV/ZIKV	0.0359
		Ctrl vs ZIKV&DENV/ZIKV	0.0035
Motor Maturity and Activity	1	Ctrl vs ZIKV	0.8736
		Ctrl vs DENV/ZIKV	0.9037
		ZIKV vs DENV/ZIKV	0.5703
		Ctrl vs ZIKV&DENV/ZIKV	0.9954
Motor Maturity and Activity	2	Ctrl vs ZIKV	0.931
		Ctrl vs DENV/ZIKV	0.786
		ZIKV vs DENV/ZIKV	0.9806
		Ctrl vs ZIKV&DENV/ZIKV	0.5223
Motor Maturity and Activity	3	Ctrl vs ZIKV	0.9499
		Ctrl vs DENV/ZIKV	0.9934
		ZIKV vs DENV/ZIKV	0.8813
		Ctrl vs ZIKV&DENV/ZIKV	0.9266
Motor Maturity and Activity	4	Ctrl vs ZIKV	0.8782
		Ctrl vs DENV/ZIKV	0.9178
		ZIKV vs DENV/ZIKV	0.5955
		Ctrl vs ZIKV&DENV/ZIKV	0.9868
Sensory Responsiveness	1	Ctrl vs ZIKV	0.7793
		Ctrl vs DENV/ZIKV	0.8363

		ZIKV vs DENV/ZIKV	0.9104
		Ctrl vs ZIKV&DENV/ZIKV	0.9586
Sensory Responsiveness	2	Ctrl vs ZIKV	0.6297
		Ctrl vs DENV/ZIKV	0.6792
		ZIKV vs DENV/ZIKV	0.9996
		Ctrl vs ZIKV&DENV/ZIKV	0.5872
Sensory Responsiveness	3	Ctrl vs ZIKV	0.7155
		Ctrl vs DENV/ZIKV	0.8441
		ZIKV vs DENV/ZIKV	0.6026
		Ctrl vs ZIKV&DENV/ZIKV	0.9463
Sensory Responsiveness	4	Ctrl vs ZIKV	0.3308
		Ctrl vs DENV/ZIKV	0.6705
		ZIKV vs DENV/ZIKV	0.9566
		Ctrl vs ZIKV&DENV/ZIKV	0.7427
State Control	1	Ctrl vs ZIKV	0.8563
		Ctrl vs DENV/ZIKV	0.3133
		ZIKV vs DENV/ZIKV	0.7228
		Ctrl vs ZIKV&DENV/ZIKV	0.1846
State Control	2	Ctrl vs ZIKV	0.9851
		Ctrl vs DENV/ZIKV	0.8625
		ZIKV vs DENV/ZIKV	0.9523
		Ctrl vs ZIKV&DENV/ZIKV	0.7583
State Control	3	Ctrl vs ZIKV	1
		Ctrl vs DENV/ZIKV	0.9981
		ZIKV vs DENV/ZIKV	0.9987
		Ctrl vs ZIKV&DENV/ZIKV	0.8699
State Control	4	Ctrl vs ZIKV	0.69
		Ctrl vs DENV/ZIKV	0.9981
		ZIKV vs DENV/ZIKV	0.543
		Ctrl vs ZIKV&DENV/ZIKV	0.7698
Orientation construct subgroup: Focus	1	Ctrl vs ZIKV	0.6065
		Ctrl vs DENV/ZIKV	0.003
		ZIKV vs DENV/ZIKV	0.0116
		Ctrl vs ZIKV&DENV/ZIKV	0.0291
Orientation construct subgroup: Visual Orientation	2	Ctrl vs ZIKV	0.2309
		Ctrl vs DENV/ZIKV	0.0213

		ZIKV vs DENV/ZIKV	0.4709
		Ctrl vs ZIKV&DENV/ZIKV	0.0385
Orientation construct subgroup: Visual Tracking	3	Ctrl vs ZIKV	0.1437
		Ctrl vs DENV/ZIKV	0.0016
		ZIKV vs DENV/ZIKV	0.1636
		Ctrl vs ZIKV&DENV/ZIKV	0.0057
Orientation construct subgroup: Auditory Orientation	4	Ctrl vs ZIKV	0.7277
		Ctrl vs DENV/ZIKV	0.1037
		ZIKV vs DENV/ZIKV	0.2172
		Ctrl vs ZIKV&DENV/ZIKV	0.1897

Supplemental Table S7. Maternal-fetal interface tissue viral loads

Group	Dam ID	Cotyledon number	Tissue viral load (copies vRNA/mg)		
			Chorionic plate	Decidua	Placenta
ZIKV	044-101	1a	0	43.3289	0
		1b	0	3.8829	0
		1c	1.833	8.725	0
		1d	0	0	0
		1e	0	0	0
		1f	0	0	0
		1g	0	9.3513	0
		1h	65.0844	0	0
		2a	731.1753	0	0
		2b	0	0	0
		2c	1066.7979	0	0
		2d	19.3648	9.5633	0.682
		2e	0	0	0
		2f	0	0	0
		2g	0	179.4231	0
		2h	0	0	0
		2i	0	6.3418	0
		2j	0	28.3216	0
		2k	0	5.0091	0
		2l	0.2786	4.4686	10.5337
	044-102	1a	0	0	0
		1b	0	0	0
		1c	0	0	0
		1d	0	0	0
		1e	0	0	0
		1f	0	0	0
		1g	0	0	0
		1h	0	0	0
		2a	0	0	0
		2b	2016.4965	0	0

		2c	0	0	0
		2d	0	0	0
		2e	0	0	0
		3a	0	0	0
		3b	0	0	0
	044-103	1a	0	0	0
		1b	0	0	0
		1c	0	0	0
		1d	0	0	0
		1e	0	0	0
		1f	0	0	0
		1g	0	0	0
		1h	0	0	0
		1j	0	0	0
		2a	0	0	0
		2b	0	0	0
		2c	0	0	0
		2d	0	0	0
		2e	0	0	0
		2f	0	0	0
		2g	NA	0	0
	044-104	1a	0	0	0
		1b	0	0	0
		1c	0	0	0
		1d	0	0	0
		1e	265.7528	0	0
		1f	0	0	0
		2a	0	0	0
		2b	0	0	0
		2c	0	0	0
		2d	0	0	0
		2e	0.1759	0	0
DENV/ZIKV	042-101	1a	4.2132	0	0
		1b	0	0	0
		1c	8.2162	NA	0
		1d	27.1164	0.172	0
		1e	NA	0	0
		1f	NA	0	0
		1g	NA	0	0
		1h	NA	0	0
		2a	NA	0	1093.0121
		2b	NA	0	59.3973
		2c	19.4595	3.095	0.248
		2d	40.694	0	13.2325
		2e		9.9504	0.3192
		2f		4.5233	0
	042-102	1a	0	0	0
		1b	0	0	0
		1c	0	0	0

		1d	NA	0	0
		1e	NA	0	0
		1f	NA	0	0
		1g	NA	0	0
		1h	NA	0	0
		1j	NA	0	0
		2a	NA	0	0
		2b	NA	0	0
		2c	NA	0	0
	042-103	1a	0	0	0
		1b	0	0	0
		1c	NA	0	0.5355
		1d	NA	0	0
		1e	NA	1.7511	0
		1f	NA	0	0
		1g	NA	0	0
		1h	NA	0	0
		2a	2.2832	0	0
		2b	NA	0	0
		2c	NA	0	0
		2d	NA	0	0
		2e	NA	0	0
		2f	NA	0	53.8472
		2g	NA	0	2.392
		2h	NA	0	0
		2i	NA	9.3524	0.0896
		2j	NA	NA	0
	042-104	1a	314.3516	0	0
		1b	1577.0798	0	19.43
		1c	0	0	0
		1d	NA	0	0
		1e	NA	0	0
		1f	NA	0	0
		2a	NA	0	0
		2b	NA	0	NA
		2c	NA	0	0
		2d	NA	0	0
		2e	NA	0	0
		2f	NA	0	0
		2g	NA	0	0
		2h	NA	0	0
		2i	NA	0	0
	042-105	1a	0	0	0
		1b	0	0	0
		1c	0	0	0
		1d	0	0	0.0501
		1e	0	0	0
		1f	0	0	0
		1g	0	0	0.2537

		1h	0	0	0.0219
		1j	0	0	1.0868
		2a	0	0	0.1007
		2b	0	22.1242	0.0954
		2c	0	0	5.5103
		2d	0	0	0
		2e	0	0	0
		2f	0	0	0
	042-106	1a	0	0	0
		1b	0	0	0
		1c	0	0	0
		1d	0	0	0
		1e	0	0	0
		1f	0	0	0
		1g	0	0	0
		1h	0	0	0
		1j	0	0	0
		2a	0	0	0
		2b	0	0	0
		2c	0	0	0
		2d	0	0	0
		2e	0	0	0
	042-107	1a	0	0	0
		1b	0	0	0
		1c	0	0	0.1904
		1d	3.6486	7.2762	0.0771
		1e	0	0	0
		1f	0	0	0
		1g	0	0	0
		2a	0	0	0
		2b	0	0	6
		2c	0	0	0
		2d	0	0	0
		2e	0	0	0
		2f	1.7155	0	14.3468
		2g	0	0	0
	042-108	1a	0	0	0
		1b	0	0	0
		1c	0	0	0
		1d	0	0	0
		1e	0	0	0
		1f	0	0	0
		1g	0	0	0
		2a	0	0	0
		2b	0	0	0
		2c	0	0	0
		2d	0	0	0
		2e	0	0	0
		2f	0	0	0

		2g	0	0	0
		2h	0	0	0

Supplemental Table S8. Maternal ZIKV infection characteristics descriptive statistics

Maternal viremia parameter	Group	Median (Range)	
Peak Viral Load	ZIKV	4.1 (3.6-6.0)	
	DENV/ZIKV	5.1 (3.9-5.8)	
Viremia Duration	ZIKV	10.0 (6.0-84.0)	
	DENV/ZIKV	44.0 (6.0-77.0)	
Placental vRNA+/total cotyledons (%)	Control	0.0% (0.0-0.0%)	
	ZIKV	0.0% (0.0-5.0%)	
	DENV/ZIKV	12.2% (0.0-43.8%)	
	ZIKV & DENV/ZIKV	2.5% (0.0-43.8%)	
Decidua vRNA+/total cotyledons (%)	Control	0.0% (0.0-0.0%)	
	ZIKV	0.0% (0.0-45.0%)	
	DENV/ZIKV	0.0% (0.0-23.1%)	
	ZIKV & DENV/ZIKV	0.0% (0.0-45.0%)	
Chorionic plate vRNA+/total cotyledons (%)	Control	0.0% (0.0-0.0%)	
	ZIKV	6.7% (0.0-25.0%)	
	DENV/ZIKV	7.1% (0.0-83.3%)	
	ZIKV & DENV/ZIKV	6.7% (0.0-83.3%)	
		**N**	**%**
Viremia duration greater than 21 days (number >21 days (%))	ZIKV	1	20%
	DENV/ZIKV	5	63%

Supplemental Table S9. Dam and infant ZIKV infection and health parameter group comparisons

Outcome	Comparison	p-value
Peak Viral Load	ZIKV vs DENV/ZIKV	0.4208
Viremia Duration	ZIKV vs DENV/ZIKV	0.463
Viremia duration greater than 21 days	ZIKV vs DENV/ZIKV	0.266
Placental vRNA+/total cotyledons	Ctrl vs ZIKV	0.4533
	Ctrl vs DENV/ZIKV	0.072
	ZIKV vs DENV/ZIKV	0.147
	Ctrl vs ZIKV&DENV/ZIKV	0.1091
Decidua vRNA+/total cotyledons	Ctrl vs ZIKV	0.4533
	Ctrl vs DENV/ZIKV	0.2202
	ZIKV vs DENV/ZIKV	0.9999

	Ctrl vs ZIKV&DENV/ZIKV	0.2322
Chorionic membrane vRNA+/total cotyledons	Ctrl vs ZIKV	0.1859
	Ctrl vs DENV/ZIKV	0.1296
	ZIKV vs DENV/ZIKV	0.6504
	Ctrl vs ZIKV&DENV/ZIKV	0.1091
CHIV+/ total cotyledons (Present %)	Ctrl vs ZIKV	0.9999
	Ctrl vs DENV/ZIKV	0.9999
	ZIKV vs DENV/ZIKV	0.9999
	Ctrl vs ZIKV&DENV/ZIKV	0.9999
Villous Stromal Calcifications (Present %)	Ctrl vs ZIKV	0.4286
	Ctrl vs DENV/ZIKV	0.9999
	ZIKV vs DENV/ZIKV	0.2364
	Ctrl vs ZIKV&DENV/ZIKV	0.5286
Placental weight	Ctrl vs ZIKV	0.3123
	Ctrl vs DENV/ZIKV	0.7989
	ZIKV vs DENV/ZIKV	0.0745
	Ctrl vs ZIKV&DENV/ZIKV	0.8557
Vasculopathy (present %)	Ctrl vs ZIKV	0.9999
	Ctrl vs DENV/ZIKV	0.9999
	ZIKV vs DENV/ZIKV	0.9999
	Ctrl vs ZIKV&DENV/ZIKV	0.4500
Infarctions/total cotyledons	Control	0.7715
	ZIKV	0.3949
	DENV/ZIKV	0.67
	ZIKV & DENV/ZIKV	0.4289
Birthweight	Ctrl vs ZIKV	0.22
	Ctrl vs DENV/ZIKV	0.9174
	ZIKV vs DENV/ZIKV	0.1266
	Ctrl vs ZIKV&DENV/ZIKV	0.6353
1st month growth trajectory	Ctrl vs ZIKV	0.8977
	Ctrl vs DENV/ZIKV	0.522
	ZIKV vs DENV/ZIKV	0.6908
	Ctrl vs ZIKV&DENV/ZIKV	06295
Apgar score: 1 min	Ctrl vs ZIKV	0.8641
	Ctrl vs DENV/ZIKV	0.9993
	ZIKV vs DENV/ZIKV	0.8194
	Ctrl vs ZIKV&DENV/ZIKV	0.8145
Apgar score: 5 min	Ctrl vs ZIKV	0.8941
	Ctrl vs DENV/ZIKV	0.8674

	ZIKV vs DENV/ZIKV	0.9999
	Ctrl vs ZIKV&DENV/ZIKV	0.5849
Apgar score: 10 min	Ctrl vs ZIKV	0.9443
	Ctrl vs DENV/ZIKV	0.8307
	ZIKV vs DENV/ZIKV	0.9684
	Ctrl vs ZIKV&DENV/ZIKV	0.5849

Supplemental Table S10. Fetal growth parameter trajectories

Groups in comparison	Fetal and placental parameter	Group	Slope	95% CI Slope	p-value*
Controls, ZIKV, DENV/ZIKV	Biparietal diameter (BPD) z-score	Control	-0.0008	-0.0014--0.0003	0.0036
		ZIKV	0	-0.0005-0.0006	0.8886
		DENV/ZIKV	-0.0003	-0.0007-0.0001	0.124
	Femur length z-score	Control	-0.0008	-0.0023-0.0008	0.3264
		ZIKV	0.0007	-0.0009-0.0022	0.3757
		DENV/ZIKV	-0.0011	-0.0023-0.0001	0.0612
	Abdominal circumference z-score	Control	-0.0004	-0.0012-0.0003	0.2347
		ZIKV	0.0005	-0.0003-0.0012	0.2225
		DENV/ZIKV	-0.0003	-0.0008-0.0003	0.3331
	Head Circumference (HC) z-score	Control	-0.0001	-0.0005-0.0004	0.7939
		ZIKV	0.0006	0.0001-0.0011	0.018
		DENV/ZIKV	0	-0.0004-0.0003	0.8462
	BPD/Femur (log-scale)	Control	-0.0029	-0.004--0.0017	<0.0001
		ZIKV	-0.0026	-0.0036--0.0016	<0.0001
		DENV/ZIKV	-0.0026	-0.0035--0.0017	<0.0001
	HC/Femur (log-scale)	Control	-0.0018	-0.0029--0.0008	<0.0007
		ZIKV	-0.0018	-0.0027--0.0008	0.0002
		DENV/ZIKV	-0.0021	-0.0029--0.0012	<0.0001
Controls, ZIKV&DENV/ZIKV animals combined	Biparietal diameter z-score	Control	0.0008	-0.0014--0.0003	0.0036
		ZIKV & DENV/ZIKV	0.0002	-0.0005-0.0001	0.2627
	Femur length z-score	Control	0.0008	-0.0023-0.0008	0.3289
		ZIKV & DENV/ZIKV	0.0005	-0.0014-0.0005	0.3263
	Abdominal circumference z-score	Control	0.0004	-0.0012-0.0003	0.2382
		ZIKV & DENV/ZIKV	0	-0.0004-0.0004	0.9803
	Head Circumference z-score	Control	0.0001	-0.0005-0.0004	0.7915
		ZIKV & DENV/ZIKV	0.0002	-0.0001-0.0005	0.2089
	BPD/Femur (log-scale)	Control	0.0029	-0.0041--0.0017	<0.0001
		ZIKV & DENV/ZIKV	0.0026	-0.0034--0.0018	<0.0001
	HC/Femur (log-scale)	Control	0.0018	-0.0028--0.0008	<0.0007
		ZIKV & DENV/ZIKV	0.0019	-0.0026--0.0012	<0.0001

*comparison to normative fetal growth parameters in a *Z*-score analysis, where slope = 0

Supplemental Table S11. Fetal growth trajectory group comparisons

Groups in comparison	Fetal and placental parameter	Comparison	p-value
Group comparison: control vs ZIKV vs DENV/ZIKV	Biparietal diameter (BPD) *z*-score	Control vs ZIKV	0.0308
		Control vs DENV/ZIKV	0.154
		ZIKV vs DENV/ZIKV	0.3023
	Femur length *z*-score	Control vs ZIKV	0.1871
		Control vs DENV/ZIKV	0.7385
		ZIKV vs DENV/ZIKV	0.0674
	Abdominal circumference *z*-score	Control vs ZIKV	0.0891
		Control vs DENV/ZIKV	0.7021
		ZIKV vs DENV/ZIKV	0.1202
	Head Circumference (HC) *z*-score	Control vs ZIKV	0.0585
		Control vs DENV/ZIKV	0.9345
		ZIKV vs DENV/ZIKV	0.046
	BPD/Femur(log-scale)	Control vs ZIKV	0.6893
		Control vs DENV/ZIKV	0.619
		ZIKV vs DENV/ZIKV	0.9673
	HC/Femur(log-scale)	Control vs ZIKV	0.892
		Control vs DENV/ZIKV	0.6021
		ZIKV vs DENV/ZIKV	0.5059
Group comparison: control vs ZIKV & DENV/ZIKV	Biparietal diameter *z*-score	Control vs ZIKV & DENV/ZIKV	0.0531
	Femur length *z*-score	Control vs ZIKV & DENV/ZIKV	0.7362
	Abdominal circumference *z*-score	Control vs ZIKV & DENV/ZIKV	0.3164
	Head Circumference *z*-score	Control vs ZIKV & DENV/ZIKV	0.3691
	BPD/Femur(log-scale)	Control vs ZIKV & DENV/ZIKV	0.584
	HC/Femur(log-scale)	Control vs ZIKV & DENV/ZIKV	0.7538

Supplemental Table S12. Maternal-fetal interface tissue descriptive statistics

	Group	Median (range) or % for Absent/present
CHIV+/ total cotyledons (Present %)	Control	0.0% (0/4)
	ZIKV	0.0% (0/4)
	DENV/ZIKV	12.5% (1/8)
	ZIKV & DENV/ZIKV	8.3% (1/12)
Villous Stromal Calcifications (Present %)	Control	100.0% (4/4)
	ZIKV	50.0% (2/4)
	DENV/ZIKV	87.5% (7/8)
	ZIKV & DENV/ZIKV	75% (9/12)
Placental weight (g)	Control	117 (107-145)
	ZIKV	129 (120-173)
	DENV/ZIKV	120 (95-140)
	ZIKV & DENV/ZIKV	120 (95-1733)
Vasculopathy (present %)	Control	25.0% (1/4)
	ZIKV	0.0% (0/4)
	DENV/ZIKV	12.5% (1/8)
	ZIKV & DENV/ZIKV	8.3% (1/12)
Infarctions/total cotyledons	Control	9.2% (0.0-45.5%)
	ZIKV	21.6% (0.0-40.0%)
	DENV/ZIKV	25.8% (0.0-53.3%)

	ZIKV & DENV/ZIKV	25.0% (0.0-53.3%)

Supplemental Table S13. Apgar score descriptive statistics

	Group	Mean	95% CI
Apgar score: 1 min	Ctrl	5.3	3.3-7.3
	ZIKV	6	4.3-7.7
	DENV/ZIKV	5.4	4.1-6.6
	ZIKV & DENV/ZIKV	5.6	4.6-6.6
Apgar score: 5 min	Ctrl	6.7	4.7-8.7
	ZIKV	7.3	5.5-9.0
	DENV/ZIKV	7.3	6.0-8.5
	ZIKV & DENV/ZIKV	7.3	6.3-8.2
Apgar score: 10 min	Ctrl	8.3	6.3-10.3
	ZIKV	8.8	7.0-10.5
	DENV/ZIKV	9	7.8-10.2
	ZIKV & DENV/ZIKV	8.9	7.9-9.9

Supplemental Table S14. Infant weight gain descriptive statistics

Group	Birthweight (week 0) mean	Birthweight 95% CI	1st month weight gain trajectory	1st month weight gain trajectory 95% CI
Control	0.48	0.43-0.54	0.02	0.005-0.0352
ZIKV	0.53	0.48-0.58	0.022	0.008-0.0249
DENV/ZIKV	0.48	0.44-0.52	0.026	0.0150-0.0369
ZIKV&DENV/ZIKV	0.5	0.47-0.53	0.024	0.016-0.0327

Supplemental Table S15. Neonatal body fluid viral loads

Infant ID	Treatment group	Age (days)	ZIKV vRNA copies/ml in plasma	ZIKV vRNA copies/ml in urine
042-504	DENV/ZIKV	0	0	NA
042-502	DENV/ZIKV	0	0	NA
042-501	DENV/ZIKV	3	0	NA
042-503	DENV/ZIKV	0	0	in process
042-507	DENV/ZIKV	0	0	NA
042-505	DENV/ZIKV	0	0	in process
042-508	DENV/ZIKV	0	0	0
042-506	DENV/ZIKV	0	0	NA
044-504	ZIKV only	0	0	0
044-503	ZIKV only	0	0	0
044-502	ZIKV only	0	0	0
044-501	ZIKV only	0	0	0
044-509	ZIKV only	7	0	NA

Not available (NA)
